# The Importance of RNA-Based Vaccines in the Fight against COVID-19: An Overview

**DOI:** 10.3390/vaccines9111345

**Published:** 2021-11-17

**Authors:** Bruna Aparecida Souza Machado, Katharine Valéria Saraiva Hodel, Larissa Moraes dos Santos Fonseca, Luís Alberto Brêda Mascarenhas, Leone Peter Correia da Silva Andrade, Vinícius Pinto Costa Rocha, Milena Botelho Pereira Soares, Peter Berglund, Malcolm S. Duthie, Steven G. Reed, Roberto Badaró

**Affiliations:** 1 SENAI Institute of Innovation (ISI) in Health Advanced Systems (CIMATEC ISI SAS), University Center SENAI/CIMATEC, Salvador 41650-010, Brazil; katharine.hodel@fieb.org.br (K.V.S.H.); larissa.fonseca@fieb.org.br (L.M.d.S.F.); breda@fieb.org.br (L.A.B.M.); leone@fieb.org.br (L.P.C.d.S.A.); vinicius.rocha@fieb.org.br (V.P.C.R.); milena.soares@fieb.org.br (M.B.P.S.); badaro@fieb.org.br (R.B.); 2 Gonçalo Moniz Institute, Oswaldo Cruz Foundation (IGM-FIOCRUZ/BA), Salvador 40296-710, Brazil; 3 HDT Bio, 1616 Eastlake Ave E, Seattle, WA 98102, USA; peter.berglund@hdt.bio (P.B.); malcolm.duthie@hdt.bio (M.S.D.); steven.reed@hdt.bio (S.G.R.)

**Keywords:** RNA vaccines, mRNA vaccines, saRNA vaccines, COVID-19, SARS-CoV-2

## Abstract

In recent years, vaccine development using ribonucleic acid (RNA) has become the most promising and studied approach to produce safe and effective new vaccines, not only for prophylaxis but also as a treatment. The use of messenger RNA (mRNA) as an immunogenic has several advantages to vaccine development compared to other platforms, such as lower coast, the absence of cell cultures, and the possibility to combine different targets. During the COVID-19 pandemic, the use of mRNA as a vaccine became more relevant; two out of the four most widely applied vaccines against COVID-19 in the world are based on this platform. However, even though it presents advantages for vaccine application, mRNA technology faces several pivotal challenges to improve mRNA stability, delivery, and the potential to generate the related protein needed to induce a humoral- and T-cell-mediated immune response. The application of mRNA to vaccine development emerged as a powerful tool to fight against cancer and non-infectious and infectious diseases, for example, and represents a relevant research field for future decades. Based on these advantages, this review emphasizes mRNA and self-amplifying RNA (saRNA) for vaccine development, mainly to fight against COVID-19, together with the challenges related to this approach.

## 1. Introduction

Vaccines are considered one of the most effective strategies to prevent, control, and eliminate infectious diseases [1,2]. The emergence of the new coronavirus, the severe acute respiratory syndrome coronavirus 2 (SARS-CoV-2), the causative agent of the coronavirus disease of 2019 (COVID-19) [3], has highlighted the importance of vaccination for global health [4]. The virus was first reported in December 2019 in the city of Wuhan in China and, by the end of October 2021, its rapid spread caused the infection of [5] nearly 245 million people worldwide, leading to major economic and social impacts [6,7]. The high transmissibility of SARS-CoV-2 can be attributed to its unique characteristics, mainly human-to-human transmission that occurs by various mechanisms, even from asymptomatic carriers [8,9,10]. Although most COVID-19 patients present mild symptoms such as dry cough, fever, and fatigue [11], the hospitalization and mortality rates of the disease are higher when compared to those of diseases caused by other respiratory viruses, such as influenza [12,13]. The sheer number of patients and the rapid progression to life-threatening conditions has resulted in devastating overloads of health care systems in numerous parts of the world [14].

SARS-CoV-2 is a single-chain positive-sense RNA virus of the *Betacoronavirus* genera. The SARS-CoV-2 viral genome has 29.8 kilobases, with a G+C content of less than 40%, and is composed of six large open reading frames (ORFs) common to coronaviruses and two untranslated regions (UTRs) at the 5′ and 3′ ends [15]. Four structural proteins—membrane (M), envelope (E), spike (S), and nucleocapsid (N)—and sixteen non-structural proteins (nsp1-16) form the RNA genome of SARS-CoV-2 [16]. Among them, the S glycoprotein is an important target of therapies since it is responsible for entry into host cells via its interaction with the angiotensin-converting enzyme 2 (ACE2) cell receptor [17,18].

Early sequencing of the SARS-CoV-2 genome allowed for the prompt determination of its sequence identity/similarity with the Middle East Respiratory Syndrome Coronavirus (MERS-CoV) and SARS-CoV (both previously responsible for concerning outbreaks), and routine sequencing has facilitated the identification of new mutated SARS-CoV-2 variants-of-concern [19]. Numerous SARS-CoV-2 variants-of-concern have been identified, most notably, the B.1.1.7 (known as 501Y.V1), B.1.351 (known as 501Y.V2), and P.1 (known as 501Y.V3) variants that were first detected and identified in the United Kingdom, South Africa, and Brazil, respectively [20,21]. On May 31, 2021, the WHO (World Health Organization) decided to simplify the names of these variants-of-concern with Greek letters. Therefore, these four variants-of-concern are now called Alpha, Beta, Gamma, and Delta, respectively [22]. Variants-of-interest, with the potential to rise in status to variants-of-concern, continue to emerge. Sequencing of the SARS-CoV-2 genome patients has allowed rapid advances in basic research as well as product development, most notably with innovation in vaccine development [23,24,25,26].

International efforts to end the current pandemic have been unprecedented in terms of resource allocation, scientific focus, and the pace of innovation [27]. Given the potential to provide the population with the necessary immunity against the virus, the widespread use of a safe and effective vaccine has become the primary goal for controlling the SARS-CoV-2 pandemic [28]. Since the beginning of the pandemic, more than 100 clinical trials of COVID-19 vaccine candidates have been conducted, involving over 150 research groups [29]. The development of vaccines for COVID-19 has been supported by significant financial investment; for example, the U.S. government has provided more than USD 10.5 billion to vaccine companies to accelerate the delivery of their products [30]. Companies have developed vaccine candidates across a variety of technological platforms, including virus-like particle, recombinant protein, inactivated virus, live attenuated virus, viral vector (replicating and non-replicating), and nucleic acid (DNA and RNA) approaches [31,32].

RNA-based vaccines were among the first to emerge and have become prominent in national immunization programs. RNA vaccine technology builds on the central dogma of molecular biology, in which messenger RNA (mRNA) is the intermediate step between the translation of the encoding DNA and the production of its respective protein. It is a technology that enables the carriage of genetic information directly into the cell, allowing endogenous protein expression instead of administering protein (antigen) as an exogenous entity such as killed or defined subunit platforms [33]. Moreover, due to its capacity to activate various pattern-recognition receptors, RNA can be very immunogenic [34]. Another logistical advantage of RNA-based vaccines is that the RNA can be produced in a cell-free environment by in vitro transcription (IVT), removing the need for cultured cells in the manufacturing process and removing the associated quality and safety issues associated with them [35]. In this way, it is possible to perform simple downstream purification to provide rapid and cost-effective manufacturing relative to other vaccine platforms [36]. Several other advantages, such as scalability, flexibility in manipulating antigens of interest, and the induction of both cellular and humoral immunity [31,37], are inherent to RNA vaccines. These characteristics previously enabled RNA-based vaccines to be evaluated against non-infectious diseases such as cancer and allowed their manufacturers to rapidly respond to emerging infectious agents such as SARS-CoV-2 [38]. For this reason, RNA-based vaccines have become attractive in the pandemic situation. Therefore, considering the potential presented by RNA-based vaccines, in this review, we provide a brief history and evaluate RNA-based vaccines within the context of the COVID-19 pandemic, describing the prospects and challenges related to their use for immunization of large populations against SARS-CoV-2.

## 2. A Brief History of RNA-Based Vaccines

As with other platforms, the development of RNA-based vaccines involves antigen discovery and analysis of the nucleotide sequence that will be translated into the protein of interest. For RNA-based vaccines, screening of modified nucleotides can optimize expression, as can the selection of an appropriate method [39]. Various methods have been used, such as modified nucleosides and the development of nanoparticles capable of stabilizing the RNA and/or improving its cellular uptake and, consequently, improving the overall bioavailability of the RNA cargo [40].

Studies that served as a basis for the development of synthetic RNA vaccines were reported from the end of the twentieth century and were motivated by the 1961 discovery of mRNA [41]. An important milestone for the use of RNA in the pharmaceutical industry occurred in 1989, when researchers from Vical Incorporated and the Salk Institute demonstrated that mRNA introduced by a liposomal nanoparticle could transfect different types of eukaryotic cells [42]. In 1990, Wolff et al. [43] reported that the injection of mRNA without a protective complex could induce protein expression within a few days. The first studies of mRNA and self-amplifying (saRNA) as vaccines demonstrated cell-mediated and humoral-adaptive immune responses, respectively, against the influenza A virus [44,45]. This was followed by studies of mRNA as immunotherapies focused on oncology with in vitro and in vivo assays, using both protected or unprotected (“naked”) mRNA [46,47,48]. It is important to note that the basic difference between these two types of RNA is associated with the number of replications and, consequently, the expression of the antigen. By presenting additional sequences in the coding region, saRNA has a self-amplification mechanism, which can result in an increase in the transcription process when compared to mRNA [49]. In addition, mRNA or saRNA are produced by IVT and, besides the advantages mentioned above, the RNA sequence can be easily modified to improve the protein synthesis, such as the addition of a 30 poly(A) tail, a capping approach, and methylated nucleosides or pseudouridine [50].

The amount of protein (antigen) expressed is directly related to the content of mRNA that can enter the intracellular environment. The presence of well-defined mRNA in the cytosol allows the presentation of exogenous and endogenous antigens to occur and can provide activation of the immune system via different pathways [51,52]. However, it has been shown that naked mRNA is more susceptible to enzymatic hydrolysis (especially by omnipresent ribonucleases), which can directly compromise its potency; since any degradation in its structure can result in the incomplete expression of the antigen [53,54]. After this discovery, different studies were conducted to develop delivery vehicles for the mRNA that would protect the molecule from degradation and improve the induction of the immune system [55,56,57]. Thus, several in vivo studies using different types of delivery vehicles have been performed in an attempt to develop a safe and effective RNA vaccine [58,59]. However, some strategies have demonstrated a lack of immunogenicity in primates and humans in contrast to small animal models (also called a “primate barrier”), making the choice of the delivery vehicle an important bottleneck in the clinical use of RNA vaccines [60]. Recently, the use of lipid nanoparticles (LNPs) as a delivery system has been the main focus for RNA vaccine development [61]. In 2007, de Jong et al. [62] demonstrated that LPN-encapsulated antigen can induce a stronger immune response and enhance immune efficacy. Since then, the importance of this delivery system in the development of safe and effective vaccines in different routes of administration has been reported, directly contributing to the advancement in the use of mRNA vaccines.

These studies were of great importance in establishing benchmarks for further evaluation and, consequently, assisting in the process of optimizing these vaccines, providing essential safety and immunogenicity, and providing a basis for the production of RNA vaccines in accordance with good manufacturing practices [63,64]. In this context, the most important innovations in RNA-based vaccine technology in recent years have been directed toward the use of optimized RNA sequencing, the application of methods that allow large-scale cGMP production, and the development of efficient and safe materials for RNA delivery [38,65].

### 2.1. RNA Vaccines against Cancer

RNA-based cancer vaccines have been designed to express tumor-associated antigens, resulting in the stimulation of T-cell-mediated immune responses [66]. Acute myeloid leukemia, brain cancer, colorectal cancer, liver metastases, esophagus cancer, glioblastoma, prostate cancer, and melanoma are all examples of clinical conditions targeted by RNA-based cancer vaccine candidates [67], with most applied in a therapeutic rather than a prophylactic manner [68,69]. To date, most RNA-based cancer vaccines have used non-replicating mRNA technology [70,71]. One of the first phase I/II studies involved the direct injection of mRNA into melanoma patients: while a 200-microgram injection of naked mRNA was safe, clinical efficacy was not demonstrated [72]. Soon after, the same group published a phase I/II trial of a protamine-protected mRNA vaccine in metastatic melanoma patients (NCT00204607), demonstrating that protamine-protected mRNA was not only safe but generated more promising clinical efficacy [73]. These trials thereby addressed the reported weakness of RNA based-vaccines being easily degraded by omnipresent ribonucleases and demonstrated the importance of the delivery format in related outcomes, prompting research regarding novel encapsulation/delivery strategies, such as the use of biopolymers, liposomes, or dendritic cells (DCs) [65,74].

Before studies in humans, in vitro and in vivo studies using DCs electroporated with mRNA assessed their ability to trigger potent immune responses against tumor antigens [47]. Boczkowski et al. showed the capacity of RNA-pulsed DC-based vaccines to reduce lung metastases in rats. Early human trials to evaluate mRNA delivery used monocyte-derived DCs transfected ex vivo with antigen-encoding mRNA by electroporation to provide a cell-based vaccine approach, with the cells being re-infused into patients [75]. Since then, many phase I/II clinical trials using this delivery strategy have been published, with prostate cancer, melanoma [76], B-cell lymphoma [77], adenocarcinoma [78], and pancreatic cancer [79] being among the examples of the therapeutic targets.

The RNActive^®^ vaccine platform (WO2002098443, WO2012019780), designed by CureVac, uses an mRNA complex with protamine and naked mRNA, where protamine assists in the process of inducing cellular immunity [80]. Furthermore, it is important to note that the ability to express the antigen within this complex is directly associated with the relationship between the mRNA and the protamine [81]. The platform has proven to be highly versatile, allowing the translation of different antigens of interest. Two cancer vaccines based on this technology have undergone phase 1/2 clinical trials: CV-9104, tested in patients with castration-resistant prostate cancer (NCT01817738) [82] and CV-9201, tested in patients with non-small-cell lung carcinoma (NCT00923312) [83]. Table 1 presents examples of clinical trials involving RNA-based vaccines against different cancers.

### 2.2. RNA Vaccines against Non-Infectious Diseases

In addition to the applications in cancer and non-infection diseases, it is important to mention that RNA-based vaccines also have a great potential to be applied to the treatment and prophylaxis of non-infectious diseases, such as autoimmune and allergic diseases [91,92]. These diseases, while apparently diverse, share a common characteristic of an undesired and inappropriate immune response. Therefore, in a different way than the approach used for non-infectious disease and cancer, which is based on the principle that RNA, together with its formulation, provides immune stimulations of T-cell and antibody responses [65], in the case of non-infectious diseases, the goal is to suppress an immune response.

Companies specializing in therapeutics with mRNA have sought solutions to a wide range of health conditions. When it comes to autoimmune disorders, previous studies have shown that mRNA therapy has a high potential application in the treatment of these diseases [93,94,95]. It is important to note that there are over 100 distinct autoimmune disorders, and they are highly complex, where for each one, there may be a different treatment approach [96]. In general, for autoimmune disease applications, an mRNA-based vaccine acts by suppressing antigen-specific immune responses [91]. A recent study performed by BioNTech RNA Pharmaceuticals researchers [91] described the disease-suppressing effects of a non-inflammatory mRNA vaccine in mice models of multiple sclerosis. It was demonstrated that the delivery of the autoimmune target antigen by the mRNA vaccine candidate into antigen-presenting cells in the lymph nodes resulted in the prevention of disease symptoms, a reduction in the disease progression, and the restoration of motor functions [91]. This study highlights that mRNA therapy has the potential to treat autoimmune diseases by increasing immune cell tolerance and consequently reducing damage without jeopardizing the immune system functions. Another important approach for mRNA-based vaccines in the autoimmune disease context relies on the application of mRNA to encode the immunomodulation of signal molecules such as cytokines. Veiga et al. demonstrated the application of mRNA encoding IL-10 as an alternative to traditional recombinant protein therapies in inflammatory bowel diseases. The expression of IL-10 in target cells resulted in a significant decrease in pathological symptoms and in the severity of intestinal inflammation. It is important to mention Moderna’s potential mRNA medicine, mRNA-6231, which was designed to trigger peripheral tolerance pathways to restore immune homeostasis and reduce autoimmune pathology by buffering autoimmune activation. mRNA-6231 encodes for IL-2, which mutein designs to activate and expand regulatory T cells, buffering the immune response [97]. The clinical trial to evaluate the safety and tolerability of mRNA-6231 is underway (NCT04916431).

When it comes to RNA-based vaccine applications for allergic diseases therapy, in contrast to cancer or autoimmune diseases applications, vaccination against allergy does not involve the administration of self-antigens [96]. It is generally accepted that allergic reactions are triggered by recurring exposure to allergens that will lead to the production of allergen-specific IgE antibodies and the subsequent activation of inflammatory cell responses by allergen–IgE immune complexes [98]. Therefore, allergic diseases are treated with allergen-specific immunotherapy since this is the treatment that can alter the immunological basis of allergic diseases with long-term effects, and no preventive vaccination against type I allergy is available [99,100,101]. mRNA-based vaccines are studied as an alternative for allergen-specific immunotherapy since, through this technology, it is possible to deliver the allergen in a pure form and in lower doses, therefore decreasing the risk of anaphylactic side effects caused by pre-existing IgE and the occurrence of the production of any novel allergen-specific to IgE. Roesler et al. demonstrated that mRNA vaccine expression of important 29 allergens could protect the induction of IgE in animal models. In this study, the benefits of mRNA vaccination were also seen in the downregulation of inflammatory lung parameters [102]. Moreover, Hattinger et al. [103] demonstrated that vaccination with mRNA vaccines was responsible for preventing an allergen-specific response by immunomodulating the TH2-type response by suppressing TH2 cytokines, eosinophils, and IgE expression, and increasing TH1-type parameters.

Although mRNA vaccines were in the spotlight during the COVID-19 pandemic, it is important to note that much work has already been completed by using this technology platform in the treatment and prophylaxis of non-infectious diseases. Such studies demonstrate the high potential of RNA vaccine application.

### 2.3. RNA Vaccines against Infectious Diseases

In addition to the push for RNA-based vaccines to fight cancer, in recent years, the use of these platforms has gained prominence against infectious agents, especially against emerging infectious diseases such as those caused by the Zika virus, *Zaire Ebolavirus*, and coronavirus [49]. In general, RNA-based vaccines against pathogens are developed through four main steps: (1) construction of an optimized sequence (capable of enhancing immunogenicity) of antigen-encoding mRNA based on the selected antigen(s) of the target pathogen; (2) determination of the delivery material, in either the presence or absence of adjuvant molecules, and influenced by the route of administration; (3) demonstration of the in vivo expression of the encoded antigen); and (4) evaluation of immune induction [67,104]. Unlike the predominance of the conventional mRNA approach against cancer vaccines, saRNA technology has been more widely evaluated against infectious diseases [105].

**Figure 1:**
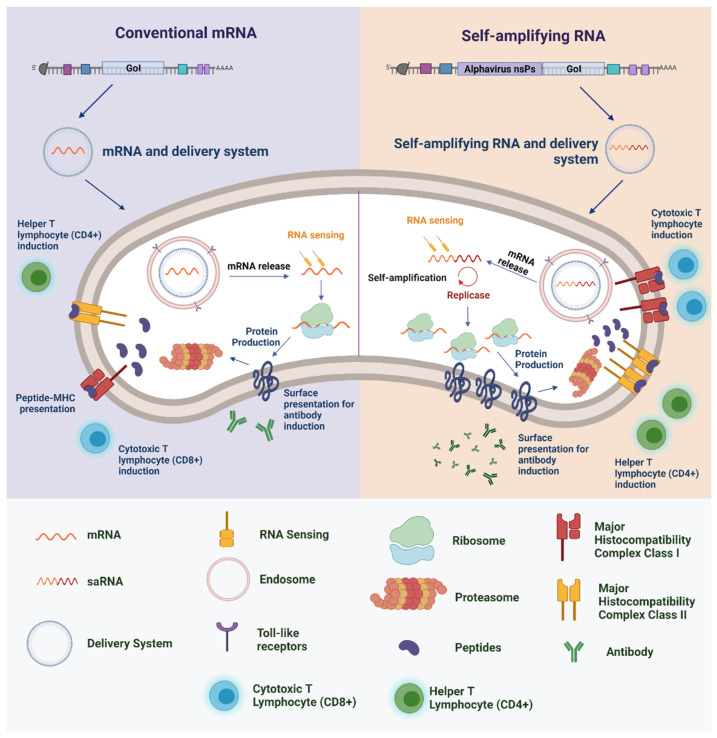
Overview of mRNA and saRNA-based vaccine mechanisms for protein production. Adapted from Maruggi et al. [104]. GoI, gene of interest; MHC, major histocompatibility complex; nsPs, nonstructural proteins. Created with BioRender.com (accessed on 30 September 2021).

saRNA replicons are created by replacing the structural genes of, typically, an alphavirus (such as Semliki Forest virus (SFV), Sindbis virus (SINV), or Venezuelan equine encephalitis virus (VEEV) with the gene for the antigen of interest. It is important to note that the absence of endogenous viral structural genes in the replicons means that the production of infectious virions or virus-like vesicles in individuals after vaccination is negated, increasing the safety profile when compared to vaccine technologies such as attenuated virus [104]. When delivered into the cytoplasm of target cells, saRNA becomes capable of amplifying the mRNA to express the target antigen at very high levels [104,106,107]. Through this self-amplification system, it has already been estimated that 200,000 copies of RNA can be made from a single saRNA molecule, resulting in higher levels of protein expression relative to those achieved by conventional mRNA (Figure 1) [108]. Thus, vaccines based on saRNA technology can induce high levels of immunity even when administered in low amounts [109].

RNA-based vaccines have been used to deliver bacterial and parasite genes but, except for vaccine candidates for *Chlamydia trachomatis* [110], most of these remain in pre-clinical or early clinical stages of development (e.g., those against the protozoan *Toxoplasma gondii* [111], *Plasmodium* [112], and *Leishmania donovani* [113]). Recently, Raj et al. [114] developed an RNA vaccine for the expression of the glutamic-acid-rich protein (PfGARP) of *Plasmodium falciparum* and showed that it induced antibody formation in in vitro assays and in a non-human primate challenge model. Maruggi et al. [115] reported that saRNA vaccines based on bacterial antigens from Group A (GAS) and Group B (GBS) *Streptococci* induced protective efficacy in mice through the induction of functional antibodies.

RNA-based vaccines have advanced further in the context of viral infections, most notably for SARS-CoV-2 but also including other respiratory viruses (SARS-CoV-1) [116], insect-transmitted viruses (Zika, dengue, and Chikungunya) [117,118,119], animal-transmitted viruses (rabies) [120], as well as viruses transmitted by direct contact with humans or their fluids (e.g., Ebola [121], herpes (herpes simplex virus and human cytomegalovirus) [122,123], and HIV [124]) (Table 2). A pioneering study involving RNA vaccines for infectious agents was conducted by Fleeton et al. [125] against three viruses: influenza A virus, a tick-borne flavivirus (louping ill virus), and respiratory syncytial virus (RSV). In vivo experiments with a mouse model showed that the RNA vaccine encoding the envelope proteins of the three viruses afforded protection against each virus evaluated [125]. Just over a decade later, Petsch et al. [126] published important preclinical data on RNActive^®^ platform-based influenza A virus infection vaccines, demonstrating that the vaccines promoted balanced, long-lived, and protective immunity against infection with influenza A virus in both small animals (mice and ferrets) and large animals (pigs) [126]. RNA vaccines can induce protective immunity against several influenza viruses and prolong the immune responses [50,127], and RNA-based vaccines are now being considered as alternatives that could overcome the bottlenecks typically faced by the conventional influenza vaccine.

## 3. RNA Vaccines in the Context of COVID-19

The response to the COVID-19 pandemic has delivered many important milestones in vaccine development, especially for RNA-based platforms. Numerous vaccine candidates were proposed in record time, with the rapid-development process guided by the knowledge acquired from coronavirus targets that had already been used in successful vaccines for humans [137]. Almost seven months after initiating a clinical development program, the BNT162b2 vaccine produced by Pfizer in partnership with BioNTech became the first vaccine approved by United States FDA for emergency use approval (EUA) against COVID-19 [138,139]. Shortly after, Moderna’s mRNA-1273 vaccine, developed in collaboration with the National Institute Allergy and Infectious Diseases (NIAID)/National Institutes of Health (NIH), became the second COVID-19 vaccine to receive EUA in the United States [140] and represented the first approval of RNA-based vaccines.

### 3.1. The Main RNA-Based Vaccines for COVID-19

RNA-based vaccines were among the first candidates to enter preclinical and clinical development against COVID-19. According to the WHO, as of October 1, there were at least 194 preclinical studies of COVID-19 vaccine candidates, of which 24 included RNA-based vaccines [141]. These studies highlight the versatility of the RNA platform and have been characterized by the use of mRNA or saRNA, as well as the use of naked or nanostructured lipid-carrier delivery strategies (Table 3). Another important point is that the translated protein structure can also be modified through mRNA or saRNA ribonucleotides’ alteration, aiming to improve protein stability, immunogenicity, and the generation of neutralizing antibodies. Pallesen et al. [142] showed the structure-based design of S protein from MERS-CoV to generate a more efficient immunogenic, which was the Spike protein stabilized in the prefused protein. Their study demonstrated that genetic vaccination can be easily modified and adapted to a specific prophylactic use.

In general, preclinical studies involving RNA-based vaccines have demonstrated that this technology can induce both humoral and cellular immune responses, with sufficient data to support advancement to clinical development phases [143,144,145,146,147]. To evaluate the immunogenicity of COVID-19 vaccine candidates, the induction of antigen-specific immunity (both antigen-binding IgG and virus-neutralizing antibodies) has been determined in animal models such as mice and non-human primates (Table 3). Accompanying the induction of circulating antibodies, the cellular responses involving CD4+ and CD8+ T cells have also typically been evaluated.

**Table 3:** Overview of preclinical studies of RNA-based COVID-19 vaccine candidates.

Developer	Vaccine Name	Active Substance (Antigen Type) and Formulation Details	Animal Model	Main Findings and/or Conclusions Considering	Reference
CureVac AG	CVnCoV	Lipid-nanoparticle-encapsulated mRNA that encodes full-length, prefusion-stabilized SARS-CoV-2 S protein	Mice (BALB/c)	Mice: innate immune response (systemic IL-6 and IFNα and robust cellular and humoral immune responses Hamsters: protected against challenge with wild-type SARS-CoV-2 NHP: those vaccinated with 8 µg were protected from challenge with wild-type SARS-CoV-2 (Victoria/1/2020)	[146]
			Syrian Hamsters (*Mesocricetus auratus*)		
			Rhesus macaques (*Macaca mulatta*)		[148]
Arcturus Therapeutics	ARCT-021 (LUNAR-COV19)	Lipid-nanoparticle-encapsulated saRNA that encodes an alphavirus-based replicon and the SARS-CoV-2 full-length S glycoprotein (saRNA)	Mice (C57BL/6 (human ACE2 transgenic mouse model) and BALB/c)	Mice: single vaccination led to robust antibody responses Human ACE2 transgenic mice: protected from mortality and measurable infection following wild-type SARS-CoV-2 challenge	[149]
Imperial College London	COVAC1 (LNP-nCoVsaRNA)	saRNA encoding SARS-CoV-2 S protein encapsulated within a cationic liposom	Mice (BALB/c)	Mice: high cellular responses (IFN-γ production), specific IgG	[143]
Pfizer and BioNTech	BNT162b1	Nucleoside-modified mRNA that encodes immunogens derived from the S protein of SARS-CoV-2 stabilized in the prefusion conformation, formulated in lipid nanoparticle	Mice (BALB/c)	Mice: one intramuscular dose of either elicited dose-dependent antibody responses, strong CD4+, and CD8+ T-cell response NHP: protected macaques against challenge with wild-type SARS-CoV-2	[144]
	BNT162b2 (known commercially as Comirnaty)		Rhesus macaques (*Macaca mulatta*)		
HDT Bio	HDT-301 (LION/repRNA-CoV2S)	saRNA that encodes SARS-CoV-2 full S protein formulated with alipid inorganic nanoparticle (LION) emulsion	Mice (BALB/c and C57BL/6)	Mice: single immunization triggered robust IgG and Th1 cells Aged mice: induced S-specific IgG and Th1 responses NHP: antibody responses persisted for at least 70 days, neutralized SARS-CoV-2 at titers comparable to those of convalescent plasma	[145]
			Pigtail macaque (*Macaca nemestrina*)		
Moderna	mRNA-1273 (known commercially as Spikevax)	Nucleoside-modified mRNA that encodes prefusion-stabilized SARS-CoV-2 encapsulated in a lipid nanoparticle	Rhesus macaques (*Macaca mulatta*)	NHP: induced robust neutralizing activity against SARS-CoV-2, rapid protection in the upper and lower airways without pathological changes in the lung after challenge	[150]
PLA Academy of Military Sciences, Abogen and Walvax	ARCoV	Lipid-nanoparticle-encapsulated mRNA encoding the receptor-binding domain of SARS-CoV-2 S protein	Mice (BALB/c)	Mice: neutralizing antibodies against SARS-CoV-2, cellular responses (Th1 cells) Two doses conferred complete protection against the challenge (wild-type SARS-CoV-2) NHP: Th1 responses, neutralizing antibodies against SARS-CoV-2 Note: manufactured as a liquid formulation that can be stored at room temperature for at least 1 week	[147]
			Cynomolgus monkeys (*Macaca fascicularis*)		

LUNAR: Lipid-Enabled and Unlocked Nucleomonomer Agent-Modified RNA; PLA: People’s Liberation Army; Names in italics represent scientific names.

Moderna’s mRNA-1273 was the first COVID-19 vaccine candidate to enter the clinical phase in the Western hemisphere. Its rapid development was made possible by the technological advances achieved through the earlier use of the company’s platform for vaccines against other viruses (Table 2), as well as the important partnership with NAID, which previously resulted in different patent applications for vaccines for other infectious diseases, including Chikungunya (WO2017070624), Zika (WO2018151816), and Ebola (WO2017015457) [151]. Table 4 provides an overview of the clinical development of RNA-based COVID-19 vaccine candidates.

mRNA-1273 contains a nucleoside-modified mRNA that encodes the stabilized prefusion SARS-CoV-2 spike protein (S-2P) based on the originally identified Wuhan lineage virus (GenBank accession number MN908947.3) that is encapsulated in lipid nanoparticles. mRNA-1273 is administered intramuscularly in two doses scheduled 28 days apart [152]. The phase 1 clinical trial of mRNA-1273 was an open-label, dose-ranging trial that evaluated the safety, reactogenicity, and immunogenicity of the vaccine in 45 healthy adults (18 to 55 years of age) (NCT04283461). This trial started on 16 March in the United States, only 5 days after the World Health Organization (WHO) classified COVID-19 as a pandemic [153] and only 66 days after the release of the genetic sequence of SARS-CoV-2 [154]. Subsequently, a further phase 1 study included 40 healthy adults aged >56 years, stratified according to age (56 to 70 years or ≥71 years) (NCT04283461), with immunological and safety profiles similar to those reported in the phase 1 study with younger adults [155]. The phase 2 (NCT04405076) and 3 (NCT04470427) trials started in sequence on May 29 and on July 20 in adults aged 18 years and older with 600 and 30,420 participants, respectively.

Given the rapid progression, the long-term safety and durability of the humoral immune response elicited by mRNA-1273 are only now becoming clear. Available data demonstrate that circulating neutralizing and binding antibody titers at day 119 after the first vaccination (90 days after the second vaccination) are still at concentrations capable of providing protection in participants of different ages [156]. In the first phase 3 trial of mRNA-1273 (NCT04470427), the inclusion of different races or ethnic groups was reported, although each of it is 99 research centers were located in the United States. Clinical trials in various regions of the world have become a common practice in recent years, allowing the analysis of safety and efficacy data within groups from different locations [157]. Moderna then progressed to two phase 2/3 studies in participants under 18 years of age, assessing the safety, reactogenicity, and effectiveness of mRNA-1273 in adolescents 12 to <18 years old (NCT04649151) and in healthy children between 6 months and <12 years of age (NCT04796896), respectively. Again, these trials are exclusive to the United States.

Although the phase 3 trial of mRNA-1273 included patients from several risk groups for developing severe COVID-19, such as diabetics, hypertensive individuals, individuals with chronic lung disease, liver disease, significant heart disease, people with HIV, and people with severe obesity, other conditions, such as patients with chronic kidney disease, were not evaluated [158]. Thus, Broseta et al. [159] analyzed the evolution of IgG antibodies after the first dose of mRNA-1273 in 78 dialysis-dependent chronic kidney disease patients, showing that there was an increase in patients with a detectable immune response as the weeks progressed (8 weeks of evaluation), indicating that mRNA-1273 is also likely effective in this group. It was noteworthy, however, that relative to the general population, patients who received a kidney transplant had a lower response even after administration of the two doses of mRNA-1273, with this likely being associated with the use of high doses of immunosuppressants [160,161]. A single dose of mRNA-1273 was found to be more likely to induce the antibody response than BNT162b2 in solid-organ transplant recipients [162].

**Table 4:** Overview of clinical trials involving the most advanced COVID-19 RNA vaccines.

Vaccine	Trial Phase	Location (NCT Number)	Doses	Dose Level	Main Immunogenicity and/or Efficacy Findings	Main Safety Findings	Reference
mRNA-1273 (Spikevax)	1	USA	2 (28 days apart)	25 μg, 100 μg, or 250 μg	Dose-dependent responses observed. Antibody GMT of 40,227, 109,209, and 213,526 in 25 μg, 100 μg, and 250 μg recipients, respectively. After second vaccination, titers increased to 299,751, 782,719, and 1,192,154 for 25 μg, 100 μg, or 250 μg, respectively.	AEs were commonly reported at the highest doses and after the second immunization. Systemic and local AEs occurred after both injections (fatigue, chills, headache, myalgia, and injection site pain). No AEs were noted; no prespecified halting rules were met.	[163]
	2	USA		50 μg or 100 μg	The 100 μg dose induced a > binding antibody concentrations than 50 μg.	The most common solicited AEs were injection site pain, headache, and fatigue.	[164]
	3	USA		100 μg	There was 94.1% efficacy (95% CI, 89.3 to 96.8%; *p* < 0.001) for the prevention of symptomatic SARS-CoV-2 infection within two weeks of second dose.	AEs were rare and occurred with the same incidence as the placebo. The most common treatment-related AEs were fatigue, headache, and injection-site pain.	[158]
BNT162b2 (Comirnaty)	1/2	Germany (NCT04380701) USA (NCT04380701)	2 (21 days apart)	10 µg, 20 µg, 30 µg	Dose-dependent nAbs > convalescent serum. (1.7 to 4.6-fold higher in 18–55-year-olds, 1.1–2.2-fold higher in 65-to-85-year-olds. SARS-CoV-2 antigen-specific CD4+ and CD8+ T cells.	Lower incidence and severity of systemic reactions, especially in older adults (65 to 85 years). Transient local reactions and systemic events were dose-dependent, greater after the second dose.	[165]
	3	USA, Argentina, Brazil, Germany, South Africa, Turkey (NCT04368728)		30 μg	There was 95% efficacy (95% CI 90.3–97.6) evaluated 7 days after second dose. Efficacy in participants reported comorbidity of 94.7%.	16–55-year-olds experienced more systemic effects than >55-year-olds. Headache and fatigue were the most common systemic effects of local, injection-site pain.	[166]
CVnCoV	1	Germany (NCT04449276)	2 (28 days apart)	2 µg, 4 µg, 6 µg, 8 µg, or 12 µg	nAbs and IgG S protein or RBD-binding IgG two weeks after the second 12 μg dose comparable to convalescent plasma.	Dose-dependent increase in the frequency and severity of systemic AEs and local reactions; most were mild or moderate and transient. No vaccine-related SAEs were reported.	[167]
	2/3	Argentina, Belgium, Colombia, Dominican Republic, Germany, Mexico, Netherlands, Panama, Peru, and Spain (NCT04652102)		12 µg	Reported 47% efficacy versus disease.	Data not yet released.	[168]
ARCT-021 (LUNAR-COV19)	1	Singapore (NCT04480957)	1	1 μg, 5 μg, 7.5 μg, and 10 μg	nAbs increased dose, similar following one 5 μg or 7.5 μg dose. S-binding IgG titers overlapped with levels in convalescent plasma (except1 μg dose).	The 10 μg dose was associated with more local and systemic solicited AEs; 7.5 μg was well tolerated. Fatigue, headache, myalgia, chills, and fever were the most common AEs.	[169]
	2		2 (28 days apart)	3 μg and 5 μg	nAbs increased with dose. No increase in CD8 IFN-γ T cells was reported after the second dose.	Fatigue and arthralgia occurred in a single participant following the second 5 μg dose (56–80-year-old cohort).	

AEs: adverse events; GMT, geometric mean titer; nAb, SARS-CoV-2 neutralizing antibody RBD, receptor-binding domain.

With an immediate USD 400 million awarded in grants [170], Pfizer and BioNTech developed the BNT162 series of COVID-19 RNA-based vaccine candidates encapsulated in a lipid nanoparticle [171]. The BNT162 series is a group of four candidate RNA vaccines (BNT162b1, BNT162b2, BNT162a1, and BNT162c2) developed with different RNA formats and target antigens: nucleoside-modified RNA (BNT162b1 and BNT162b2), non-modified uridine RNA (BNT162a1), and saRNA (BNT162c2), all with capabilities to synthesize protein S in its prefusion conformation [172]. Based on the Wuhan lineage virus encapsulated in a lipid nanoparticle, the BNT162b1 and BNT162a1 vaccines encode a trimerized receptor-binding domain (RBD) of the S protein, while the BNT162b2 and BNT162c2 vaccines encode a full-length spike protein [171]. Two phase 1/2 umbrella trials in Germany (NCT04380701 and EudraCT 2020-001038-36) and in the United States (NCT04368728) evaluated multiple vaccines under a single trial protocol [154]. Results have been published from phase 1 trials of BNT162 vaccines in Germany [173], the United States [165,174], and China (NCT04523571) [175]. Initially, the phase 1 study conducted in Germany evaluated the four different vaccines (BNT162a1, BNT162b1, BNT162b2, and BNT162c2), with candidates BNT162b1 and BNT162b2 prioritized for further development.

Data from Sahin et al. [173] suggest that two doses of 1–50 μg of BNT162b1 could protect against COVID-19 through multiple mechanisms, including humoral and cellular responses. The trial conducted in the United States demonstrated that the cellular and humoral response induced by BNT162b1 was dose-dependent, as was the reactogenicity: the second dose of the highest concentration evaluated (100 μg) was not administered due to increased reactogenicity [174]. Phase 1 safety and immunogenicity results of BNT162b1 in younger and older Chinese adults indicated a safe profile, with AEs that were transient and resolved spontaneously or could be managed with a simple standard of care [175]. BNT162b1, however, generated a higher incidence and severity of systemic reactions than BNT162b2, particularly in older adults (65 to 85 years old), while eliciting SARS-CoV-2-neutralizing antibodies in a similar dose-dependent manner [165]. BNT162b2 was selected for expanded clinical evaluation to support an application for marketing authorization [176]. For phase 2/3 (NCT04368728), 43,548 people underwent randomization at >160 sites worldwide (including Brazil, Argentina, Turkey, and South Africa) (Table 4), with about 20% of participants reporting some comorbidity (most commonly diabetes mellitus, hypertension, or chronic lung disease). The safety and efficacy of BNT162b2 in pregnant women, an important risk group for COVID-19 [177], was not released alongside the interim analysis, but Goldshtein et al. [178] demonstrated that pregnant women (gestational age up to 5 weeks) who received a single dose of BNT162b2 had a lower risk of SARS-CoV-2 infection 28 days after immunization than unvaccinated pregnant women, and Bookstein et al. [179] demonstrated that two doses of BNT162b2 generated a humoral immune response in pregnant women at 2–40 weeks of gestation, although anti-Spike RBD IgG levels were lower than those observed in vaccinated, non-pregnant women.

The high efficacy demonstrated in the phase 3 trial of BNT162b2 supported its eagerly awaited application for emergency use authorization (EUA). On 2 December 2020, UK regulators granted EUA to BNT162b2, making it the world’s first approved COVID-19 vaccine [180]. Nine days later, the US Food and Drug Administration (FDA) also granted EUA, facilitating its approval by other regulatory agencies around the world [181]. The high efficacy observed in the phase 3 trial, however, raised questions as to whether the conditions evaluated would represent “real life” outside of clinical trials. In phase 3 studies, conditions such as the monitoring and maintaining of the cold chain required to preserve vaccine integrity and the administration of the vaccine doses within the expected interval were closely followed, preventing the occurrence of problems in maintaining the quality and stability of the vaccine [182]. However, these variables can be difficult to control as mass vaccination is implemented, which causes the results related to the level of protection given the investigational product to be overestimated in the “real-world” [183]. Normally, these conditions are only evaluated in phase 4 studies, where the wide deployment of the vaccine is considered [184]. When Chodick et al. [185] analyzed the effectiveness of BNT162b2 in adults in Israel, they found that the first dose was associated with a ~51% reduction in the risk of SARS-CoV-2 infections on days 13 to 24 after immunization and 54% efficacy against symptomatic COVID-19, a similar level to that reported after provision of the first dose in the phase 3 study [166,185]. In a prospective cohort study conducted in Mexico to evaluate the effectiveness of BNT162b2 among the high-risk group of healthcare professionals, efficacy against severe cases of COVID-19 among individuals with only a single dose was 100% [186]. Thus, the efficacy rates reported from trials have generally held up under real-world conditions (Table 5).

In addition to being the first vaccine approved within the context of the pandemic of COVID-19, BNT162b2 was the first vaccine to be licensed for use in adolescents between the ages of 12 and 15 in countries such as the United States [192], Canada [193], and Brazil [194], as well as the European Union [195]. The authorization for use in adolescents came after the release of the phase 3 study initiated in the United States that evaluated BNT162b2 in children aged 12 to 15 years (NCT04368728). The vaccine demonstrated 100% efficacy against confirmed COVID-19 and induced strong immunogenicity one month after the second dose [196]. In parallel, another phase 1/2/3 study is being conducted at over 101 sites to evaluate the safety, tolerability, and immunogenicity of BNT162b2 administered at three different dosages (10 μg, 20 μg, and 30 μg) in healthy children between 6 months and 12 years of age (NCT04816643). It is expected that, as with adults and the elderly, the worldwide population under the age of 12 will soon have access to vaccines against SARS-CoV-2.

CVnCoV, the COVID-19 vaccine candidate developed by the German company CureVac, is based on mRNA technology capable of encoding the prefusion-stabilized form of the full-length S protein of the Wuhan lineage virus (GenBank accession number YP_009724390.1) and formulated on the LNP-formulated RNActive^®^ [197]. It is important to note that there has been an alteration in the vaccine development strategy by CureVac, as a lipid carrier has replaced the protamine-based carrier that had been commonly used by the company, as presented in Table 2. This change may have been motivated by the advantages presented by the lipid vehicles, such as the ability to induce neutralizing antibodies after administration of the vaccine via standard intramuscular injection, whereas this property has only been demonstrated for protamine-based formulations since their administration without needles (a less common route), as well as by the better storage conditions of the LNPs [198,199]. The development of CVnCoV was carried out with the expectation that, because it only required refrigerated storage, it could facilitate access to low-income countries [168]. The phase 1 clinical trial of CVnCoV (NCT04449276) started in July 2020 in Germany and Belgium, following preclinical studies indicating that low doses of CVnCoV induced high titers of neutralizing antibodies and a robust cellular immune response against SARS-CoV-2 [146,148]. The antibody responses were elicited in a dose-dependent manner, and a 12 μg dose was selected for phase 2/3 clinical investigation (NCT04652102, NCT04515147, and NCT04674189) in Argentina, Peru, Panama, Belgium, Mexico, the Netherlands, as well as other European and Latin American countries [167]. Preliminary phase 3 data showed that the vaccine was only 47% effective at preventing COVID-19 [200], almost two times less effective than vaccines BNT162b2 and mRNA-1273 and below the 50% target initially set by the WHO.

The lower performance of CVnCoV relative to mRNA-1273 and BNT162b2, which use the same technological development platform, was speculatively attributed to variants-of-concern found in the countries where the study was conducted (such as the Lambda variant, circulating in different Latin American and European countries) [201,202]. However, other RNA vaccines are effective in controlling new variants-of-concern of SARS-CoV-2 [203,204,205], and the main reason for the relatively poor performance of CVnCoV may relate to either the low doses employed or the mRNA sequence used (CVnCoV uses normal uridine and is thus “unmodified” mRNA, whereas mRNA-1273 and BNT162b2 use a modified mRNA that replaces uridine with N1-methylpseudouridine). mRNA modified with N1-methylpseudouridine can result in more durable protein expression and thus longer antigen availability and may result in more robust immune system responses [206,207]. CureVac recently announced a new COVID-19 vaccine candidate called CV2CoV, a second-generation mRNA vaccine developed through a partnership with GlaxoSmithKline (GSK). This candidate is based on the analysis of potential SARS-CoV-2 variants-of-concern in multivalent vaccine formats. Recent preclinical data for CV2CoV show that it induced complete protection against variant B.1.351 in a transgenic mouse model [208].

The first RNA vaccine approved for clinical trials in China was ARCoV (CN111333704), a vaccine developed by the People’s Liberation Army (PLA) Academy of Military Sciences in collaboration with Suzhou Abogen Biosciences and Walvax Biotechnology Co. ARCoV is a lipid-nanoparticle-encapsulated mRNA vaccine encoding the RBD domain of SARS-CoV-2 (Wuhan) S protein [209]. Phase 1 and phase 2 trials evaluating the safety and immunogenicity of different doses of ARCoV in Chinese adults aged 18–59 years began in June 2020 (ChiCTR2000034112) and January 2021 (ChiCTR2100041855), respectively, while Phase 3 evaluation is being conducted in China and Mexico (NCT04847102) [210]. A major advantage of ARCoV over other RNA vaccines lies in its thermal stability as a liquid formulation that can be stored at room temperature for at least 1 week, thus facilitating distribution [147].

Vaccines based on saRNA technology also rapidly emerged. Among them, ARCT-021 (LUNAR-COV19), developed by Arcturus Therapeutics (San Diego, California, US) and Duke–NUS Medical School in Singapore, uses the STARR™ (Self-Transcribing and Replicating RNA) system in combination with the LUNAR^®^ (Lipid-Enabled and Unlocked Nucleomonomer-Agent-Modified RNA) delivery system. ARCT-021 carries a sequence that encodes alphavirus replicase and the full-length, unmodified S protein (Wuhan sequence) [211]. This system is designed to increase and extend the expression of the antigen of interest, allowing vaccination at lower doses than conventional mRNA vaccines [212]. A differentiating feature of ARCT-021 is that, unlike the other vaccines mentioned above that are distributed as frozen liquids, it is presented in a lyophilized form that negates the need for an ultra-cold chain, which needs consistent storage at extremely cold temperatures (storage at about <−70 °C) [211]. Phase 1 (NCT04480957) and phase 2 (NCT04480957) trials are summarized in Table 4. Data from 42 phase 1 and 64 phase 2 patients indicated that ARCT-021 was well tolerated in a single 7.5 μg dose regimen and in a two-dose 5 μg regimen at the concentrations shown in Table 4. Neutralizing antibody titers increased with increasing dose, in addition to triggering the T-cell response against the S protein of SARS-CoV-2. ARCT-021 at a dose of 7.5 μg also showed satisfactory tolerability, with no vaccine-related serious AEs reported [169]. These results supported the further clinical development of ARCT-021, with a phase 2 clinical trial being conducted in the United States and Singapore with 600 participants (NCT04480957) to provide guidance for a subsequent phase 3 clinical trial.

With funding support from the British Government, Imperial College London also used saRNA technology to encode the prefusion-stabilized SARS-CoV-2 S-glycoprotein (Wuhan sequence) along with RNA replicase from an alphavirus, encapsulated in a lipid nanoparticle (COVAC1; LNP-nCoVsaRNA). After preclinical results demonstrated that two doses of COVAC1 induced cellular and humoral responses [143], a phase 1 trial was initiated in the United Kingdom in June 2020 to evaluate the safety and immunogenicity of COVAC1 in 320 18–75-year-olds (ISRCTN17072692). The trial evaluated COVAC1 at doses of 0.1 µg, 0.3 µg, and 1.0 µg, administered with a 4-week interval between the first and second injection. [213]. Although efficacy data have not yet been reported, together with Morningside Venture, Imperial College London has backed the creation of VacEquity Global Health (VGH), which aims to develop and distribute the COVAC1 in the United Kingdom as well as low- and middle-income countries [214].

HDT Bio’s COVID vaccine program, HDT-301, pairs a saRNA with a lipid inorganic nanoparticle (LION) formulation. Preclinical studies have demonstrated that HDT-301 induces neutralizing antibodies as well as a Th1-mediated immune response in both mice (including aged animals) and non-human primates. The generated neutralizing antibodies persisted for at least 70 days post-vaccination at titers compared to convalescent human serum [145]. Following technology transfer to the Indian biopharmaceutical Gennova Biopharmaceuticals (Pune, India), GMP manufacture and clinical evaluation of the HGCO19 vaccine candidate (incorporating the D614G variant sequence) was initiated [215] in a phase 1/2 trial (CTRI/2021/04/032688) to assess the safety and immunogenicity of a two-dose schedule of immunization with the experimental vaccine at either 5, 10, or 25 µg in healthy seronegative Indian adults. In late August 2021, the Drugs Controller General of India approved the advancement of HGC019 to phase II/III trials after it was found to be safe, tolerable, and immunogenic during its phase 1/2 evaluation. Phase 1 trials of HDT’s saRNA alternative SARS-CoV-2 Spike sequences have been approved by the FDA in the United States and Anvisa in Brazil (NCT04844268), respectively, and are scheduled to begin soon.

### 3.2. Efficacy of RNA-Based Vaccines against SARS-CoV-2 Variants

Several analyses of both clinical trial data and samples have assessed the ability of various COVID-19 vaccines to protect or generate neutralizing antibodies against different variants-of-concern of SARS-CoV-2 [216,217,218]. It is important to mention that the spike is not the only protein to become mutated. Mutations in the ORF1ab, ORF8, and nucleocapsid protein also occur [219]. Cai et al. [220] reported a comprehensive analysis demonstrating that, in terms of efficacy, RNA-based vaccines are the most effective and are capable of reaching greater than 94% efficacy. The authors attribute this achievement to the strong immunogenicity and effective presentation of SARS-CoV-2 antigens to the immune system of these vaccines. However, with the world population still to be immunized against SARS-CoV-2, ensuring the effectiveness of vaccines against the new variants-of-concern become a major concern for health authorities and scientists [221]. Currently, most of the data relating to the efficacy of RNA vaccines against SARS-CoV-2 variants-of-concern came from laboratory studies [222,223] that, in general, reveal that the vaccines elicit lower levels of neutralizing antibodies against SARS-CoV-2 variants-of-concern than against older isolates [222,223].

The B.1.1.7 variant was demonstrated to be more infectious than the Wuhan strain, around 60%, and also more resistant to monoclonal antibodies targeting the N-terminal domain [224]. However, both Comirnaty and Spikevax vaccines remain robust against this variant, generating antibodies two-fold less efficient in neutralizing the B.1.1.7 strain [225,226]. The second relevant variant found first in South Africa, known as B.1.351, shares the B.1.1.7 mutations D614G and N501Y in the S protein, which confers to this variant’s high transmissibility. The B.1.351 variant is more resistant to neutralization by convalescent serum and by serum from Comirnaty- and Spikevax-vaccinated people by six-fold [227,228]. The P.1 mutant was first detected in Brazil and shares D614G and N501Y S mutations with the other variants. Moreover, K417N/T and E484K mutations in the S protein are also found in RBD from the B.1.351 variant [229]. The Spikevax vaccine also presented reduced effectivity in generating neutralizing antibodies against P.1 compared to the original strain [223].

It is important to highlight that researchers believe the antibodies produced still might be sufficient to protect against SARS-CoV-2 infection, or at least against severe cases of COVID-19, since they can still neutralize the virus overall [230]. Since RNA technology can be more easily adapted to new variants-of-concern, and due to the important levels of efficacy observed against previously circulating SARS-CoV-2, RNA vaccines have enormous potential against SARS-CoV-2 variants-of-concern [230]. Since RNA technology can be more easily adapted to new variants-of-concern, and due to the high levels of efficacy observed against previously circulating SARS-CoV-2, RNA vaccines have great potential against SARS-CoV-2 variants-of-concern.

### 3.3. Composition of Main RNA-Based Vaccines for COVID-19

Within a brief period between the disclosure of the genetic code of SARS-CoV-2 and the authorization for use by different regulatory agencies, a large amount of information on RNA-based vaccines has been made available, which allows for a better investigation of the characteristics and composition of each developed vaccine, as well as enabling one to assess how these factors may influence immunogenicity and, consequently, the resulting protection. Table 6 shows a comparison of the components found in the main RNA vaccines against COVID-19, considering the mRNA or saRNA aspect, type of antigen used, and the composition of their delivery platform.

Naked RNA cannot pass through the cell membrane and efficiently leak into the cytoplasm due to its intrinsic properties such as size, charge, and degradability [231,232]. Therefore, RNA vaccines require effective and safe delivery methods. Most RNA vaccines evaluated have the same antigen delivery strategy: the use of LNPs. This prominence has been achieved due to critical advances that have been made in the LNPs’ formulation processes, primarily to achieve improvements in formulation stability, which increase the ability to induce cellular and humoral immune responses, improve endosomal escape, decrease reactogenicity, and increase the efficacy of RNA delivery into the cytosolic environment [107]. In general, LPNs provide a solid lipid structure for RNA protection and are basically composed of four components: (i) cationic or ionizable lipids that act in the RNA complexation to increase cellular uptake efficiency, as well as promote endosomal escape; (ii) cholesterol, which has the function of stabilizing the nanoparticle; (iii) auxiliary phospholipids that act by stabilizing the lipoplex and promoting membrane fusion; and (iv) PEGylated lipids to reduce non-specific interactions, preventing particle aggregation [233,234].

The development of an effective LNP platform for mRNA or saRNA delivery involves a crucial step, which is the choice of cationic or ionizable lipid. Considering the RNA vaccines developed against COVID-19, ionizable lipids were used by the vast majority of developers (Table 6). This choice may be associated with the advantages that ionizable lipids possess: even though they present the same capacity to complex RNA as cationic lipids, studies have shown that their use provides more safety to the formulations [235,236]. More specifically, ionizable lipids exhibit a positively charged pKa at a low (acidic) pH, which allows anionic RNA complexation. However, it is important to highlight that, in a neutral pH environment, the charge of ionizable lipids is neutral, which may reduce possible nonspecific interactions and hence their toxicity [198].

The vaccines mRNA-1273 (Spikevax) and BNT162b2 have the molecules SM-102 and ALC-0315 as ionizable lipids, respectively, in their formulations [237]. These lipidic molecules present a biodegradable character due to the presence of ester bonds in the lipid tails, resulting in a chemical similarity [238]. However, they exhibit structural differences that may have a bearing on the immunogenicity induced by these vaccines, in addition to the efficacy of mRNA delivery [206]. ALC-0315 is also estimated to be the ionizable lipid found in the carrier system of CVnCoV [238]. According to the developers of ARCT-021, the ionizable lipid molecule used on the LUNAR^®^ platform is also biodegradable and has optimized efficiency for mRNA delivery [239] due to the ability of this lipid to be rapidly degraded under normal physiological conditions by ester bond breaking, resulting in rapid metabolization and an improved safety profile [240]. The saRNA from the COVAC1 vaccine (LNP-nCoVsaRNA) is delivered in an LNP system that is owned by Acuitas. One of the probable ionizable lipids used in this formulation is Lipid A9 [238], developed by Acuitas and described in patent document US10221127B2 (lipids and lipid nanoparticle formulations for delivery of nucleic acids), which has equivalent properties to ionizable lipids SM-102 and ALC-0315 [236]. In addition to ionizable lipids, it is important to note that the helper lipids used in the RNA vaccine formulations against COVID-19 are similar, with variations in their concentration [240]. The HDT-301 (LION/repRNA-CoV2S) COVID-19 vaccine candidate uses a delivery system based on lipid inorganic nanoparticles (LION), more specifically, nanoemulsions, where the molecule DOTAP acts as a cationic lipid capable of establishing an electrostatic association with RNA molecules [145]. Like LPNs, cationic nanoemulsions (CNE) are also considered as an important antigen delivery platform within the context of RNA vaccine development. In general, the emulsions used are typically a water-in-oil emulsion, composed of three more components in addition to the cationic lipid: (1) squalene, (2) sorbitan trioleate (or monostearate in the case of HDT-301), and (3) polysorbate 80 [61]. It is important to highlight that one of the differentials found in the LION formulation compared to conventional CNEs (cationic nanoemulsions) is the presence of superparamagnetic iron oxide (Fe_3_O_4_) nanoparticles (SPIO), which can act as the stability intensifier. Preclinical trials have shown good efficacy of CNEs in delivering antigens against HIV, RSV, and cytomegalovirus and in inducing immune responses [241]. A feature of this approach is the ability to store the carrier system (CNEs) and saRNA or mRNA in separate vials and only combine them at the time of vaccine administration [238].

In addition to the delivery system, the engineering involved in the mRNA construction process is one of the critical points for an RNA vaccine to achieve an acceptable safety and efficacy profile. Optimization of the mRNA sequence to improve the immunogenic and safety profile has been one of the main challenges in developing new vaccines. This optimization has focused on the following approaches: increasing mRNA half-life to increase the duration of protein expression and protein levels, the optimization of poly-(A)-tail length, the modification of the 5′ and 3′ untranslated regions (UTRs) to adjust immunogenicity, capping strategies to prevent rapid mRNA degradation, and the incorporation of modified nucleotides within the coding sequence [242,243,244]. Thus, even non-structural (or non-coding) elements have importance in achieving the key outcomes associated with producing a vaccine, since they can interfere with mRNA stability and translatability [245]. The incorporation of N1-methylpseudouridine (m1Ψ), RNA capping, and codon optimization were the main strategies adopted by the developers of the RNA-based COVID-19 vaccines (Table 6). The use of modified nucleotide m1Ψ has been one of the main discussions within the strategies for mRNA engineering for COVID-19 vaccines. The presence of modified uridine in the mRNA construct may promote increased immune evasion, as it can minimize the recognition of the mRNA by proteins involved in the innate immune response to exogenous mRNA, in addition to increased protein production due to its ability to facilitate the translation of the mRNA into proteins via the ribosome [246,247]. These features may increase the biological stability of the mRNA and may also reduce the reactogenicity of vaccines using this technology [248]. The low efficacy of CVnCoV compared to mRNA-1273 (Spikevax) and BNT162b2 has been mainly related to the absence of these modified uridines in the structure of the CVnCoV mRNA, since they may confer stability to the mRNA before administration [249].

The combination of the delivery system with improved properties and the optimized mRNA should promote the initiation of the translation process of the target antigen in vivo. During previous outbreaks caused by other coronaviruses, the S protein has been considered as the main antigen for the development of vaccine candidates and, similarly, all mRNA vaccines against SARS-CoV-2 were developed to induce immune responses against the S protein or the receptor-binding domain [172]. Preclinical studies with candidate vaccines against SARS-CoV-1 and MERS-CoV from different technology platforms have shown that the presence of a full-length S gene or the protein itself induces both neutralizing antibody responses and protective immunity [250,251,252], which may have been used as the basis for the widespread use of this antigen in SARS-CoV-2 vaccines, as shown in Table 6. An important optimization strategy widely used by the developers of the SARS-CoV-2 mRNA vaccines (such as Pfizer and BioNTech, CureVac, Moderna, and Imperial College London) was the use of a gene sequence with a minor change that allows for the translation of the full-length S protein with two proline (2P) substitutions (K986P and V987P mutations), leading to greater stability during pre-melting conformation of the glycoprotein. Consequently, it is estimated that this modification may lead to a greater induction of antibody formation [253]. This strategy was also based on previous lessons learned during the development of vaccines against other CoVs, such as SARS-CoV-1, MERS-CoV, and HKU1 [116,142]. Despite its importance, studies have shown that the full-length S protein may also be associated with the induction of harmful immune responses through antibody-dependent enhancement mediated by non-neutralizing antibodies [254,255,256]. This evidence may have motivated the developers of the ARCoV vaccine to use the RBD subunit of the SARS-CoV-2 S-protein as an antigen, which has been the region of the S-protein that has major neutralizing epitopes capable of inducing high titers of neutralizing antibodies and low levels of non-neutralizing antibodies when compared to the full-length S-protein [17,257,258].

Furthermore, it is important to note that the definition of the components of a vaccine has a direct bearing on the possible costs of manufacturing and making the vaccine available. One of the most important advantages of the RNA platform over conventional vaccine technologies is that its manufacture is simpler, since it does not contain steps such as cell culture, which require more time and investment [107]. Moreover, even mRNAs encoding different antigens have similar physical and chemical characteristics, which means that once the manufacturing process is established, no major adaptations in the facility are required [249]. However, it is important to note that some factors may influence the costs associated with the production of RNA vaccines for COVID-19, such as the materials required to produce the delivery system and mRNA, particularly if there is a need for the inclusion of modified nucleosides and capping in the mRNA sequence, as well as the costs with consumables, such as the use of single-use equipment. For example, the CleanCap reagent (TriLink Biotechnologies, Inc., San Diego, CA, USA) is a major cost component for the production of vaccines developed by Moderna, Pfizer and BioNTech, CureVac, and Imperial College London [259]. Within this expectation, saRNA-based vaccines are expected to have a lower production cost, since a lower concentration per dose of RNA is needed to induce the required immune response, which reduces the amount of materials needed for their manufacture.

## 4. Perspectives and Challenges Associated with COVID-19 Immunization Based on RNA Vaccines

**Figure 2:**
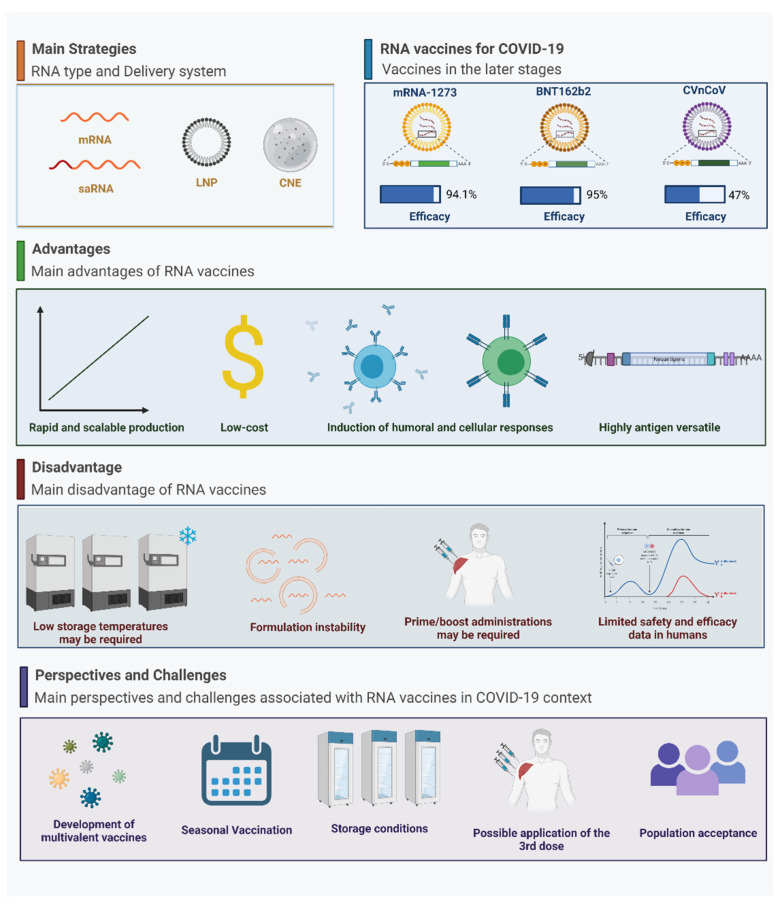
Perspectives and challenges for RNA-based vaccines for COVID-19. Created with BioRender.com (accessed on 30 September 2021).

The COVID-19 pandemic has quickly transitioned RNA vaccines from a promising platform to becoming a major tool for COVID-19 prevention and a return to normal daily routines. Following their FDA emergency use authorization (EUA), by March 2021, more than 170 million doses of RNA-based vaccines had been made available worldwide, representing 43% of the total vaccines produced [260]. Within eight months of EUA, BNT162b2 (known as Comirnaty) has been approved in almost 90 countries, while mRNA-1273 (now named Spikevax) has been approved in more than 60 countries, making these two vaccines central within national immunization programs [261]. As observed in other COVID-19 vaccines based on different technology platforms, such as the adenoviral vectors vaccines that have been widely distributed, immunization with COVID-19 RNA vaccines still faces challenges associated with mass production, extended distribution chains, sustained efficacy in the face of emergent variants-of-concern, amongst others (Figure 2).

### 4.1. Perspectives and Challenges Associated with Widespread Production and Availability

One of the main challenges associated with RNA vaccines is related to the maintenance of the immune response over time against SARS-CoV-2 through neutralizing antibody titers. In a study conducted in Israel with 4868 participants, it was observed that in the third month after administration of the second dose of the Comirnaty vaccine, IgG antibody levels decreased at a consistent rate; in the same period, the level of neutralizing antibodies decreased rapidly, with an average reduction of more than 70% from initial values [262]. Another study analyzing the same vaccine found that 3 months after the second dose, there was a reduction of up to 44.7% in antibody titers [263]. The study by Khoury et al. [264] demonstrated that the concentration of neutralizing antibodies could predict immune protection from symptomatic SARS-CoV-2 infection, where antibody decay indicates a higher probability of infection. Importantly, the decline in antibody levels may be related to the reduced effectiveness of RNA vaccines in preventing SARS-CoV-2 infection. In a recent preprint, compared to the 96% observed efficacy two weeks after receipt of both doses, the efficacy of the BNT162b2 (Comirnaty) vaccine against symptomatic COVID-19 decreased to 84% 6 months after the second dose [265]. Studies by Benotmane et al. [266] and Hall et al. [267] indicated that the provision of a third mRNA-1273 (Spikevax) dose in transplant recipients increased the humoral immune response and antibody titers. A similar result was reported by Del Bello et al. [268], who analyzed the efficacy of BNT162b2 (Comirnaty) under the same conditions. To address this issue, booster immunizations are now being recommended for some at-risk individuals in the United States and some other countries [269]. This concern is accentuated by the possibility of the emergence of new variants-of-concern with a higher morbidity and mortality rate. Thus, the development of mRNA vaccines that protect for a longer period is a major challenge for developers.

However, because new variants-of-concern usually present with mutations in the S protein gene that can reduce the affinity of neutralizing antibodies, another strategy under consideration is the use of variant-specific and/or multivalent vaccines. In this context, as discussed earlier, the manufacturing flexibility of RNA-based vaccines affords an advantage over other vaccine platforms. Moderna has adjusted its mRNA vaccine to match the Spike gene sequence of variant 501Y.V2 [270], and clinical trials are already underway (NCT04785144). Thus, a major expectation is that, like other respiratory viruses such as influenza, SARS-CoV-2 will eventually become endemic and present seasonally, which will require developers to constantly update the antigen used [271].

The countries where COVID-19 vaccines have been incorporated into the routine of the health system have had to prepare strategic planning related to their storage, transport, manipulation, stock control, and distribution, mainly because approved mRNA vaccines currently require special storage conditions [272,273]. The stability of mRNA-1273 and BNT162b2 require extremely low temperatures (between −80 to −20 °C) for distribution within their approved specifications, restricting availability to countries and regions where ultra-cool equipment is not widely available [274]. This scenario is typical of many developing countries, where the purchase of refrigeration equipment costing upwards of USD 20000 per unit is prohibitive even before considering that many communities live without continuous electrical power capable of supporting such equipment [275]. It is important to note that outside of exceptional conditions, such as the pandemic caused by SARS-CoV-2, vaccines that require a storage temperature below −20 °C are not in compliance with the requirements placed on WHO prequalification [276]. These special storage conditions can result in not only logistical and economic difficulties but can also have a direct impact on environmental issues. The study by Santos et al. [277] demonstrated the possible impacts of this special storage condition on electricity consumption and pollutant emission, where it was reported that stock maintenance of Comirnaty and Spikevax vaccines is able to emit more CO_2_ and consume more electricity compared to vaccines developed from other technologies, such as CoronaVac and Sputnik V. Thus, the development of thermostable RNA-based vaccines that do not require expensive equipment and special storage conditions and distribution chain is of importance. The use of thermostable lipid nanoparticles in the RNA system and freeze-dried techniques are considered the technological advancements that could achieve this goal [211,278].

Another important challenge is associated with the production of RNA vaccines. Biopharmaceutical companies are estimated to be able to produce up to 12 billion doses by 2021; however, to have an equitable distribution of doses among countries, especially considering the need for booster doses, this amount is not believed to be sufficient [279]. However, a point of importance is that production sites for RNA vaccines are primarily concentrated in high-income countries, necessitating that middle- and low-income countries require importation and adaptations beyond the cold chain for internal distribution [259]. Given this need, countries such as South Africa and India have sought to establish partnerships to produce RNA vaccines in their territories [280,281]. This can be reflected in the low availability of RNA vaccines to the population, where, in India, more than 85% of its population has been immunized with a vaccine based on viral vector technology [261]. In addition, according to the study by Rosa et al. [50], the production of RNA vaccines in a sustainable and cost-effective way has important challenges and bottlenecks identified in the downstream and upstream production steps, such as the use of high-cost and limited availability reagents, as well as the use of methods with low stability. In this way, it is fundamental that new production methods are proposed so that the stage of availability, commercialization, and meeting the market demand can be supplied in a quick and equitable manner.

### 4.2. Perspectives and Challenges Associated with Acceptance of COVID-19 Vaccines

Although the approval of the first RNA vaccine during the COVID-19 pandemic was a milestone, its uniqueness and rapid development led to public concerns about the safety of RNA-based vaccines [282]. This skepticism has strengthened anti-vaccine movements, raising questions about the new technology and its “real” performance [283]. Solís Arce et al. [284] showed that concerns about possible side effects are the most common reasons for the reluctance of people to receive these vaccines, despite an ever-building safety profile and regulatory scrutiny that skepticism has brought. Public perceptions may be incorrect; through a real-world safety and reactogenicity study, Mathioudakis et al. [285] demonstrated that recipients of mRNA vaccines reported milder and less frequent systemic side effects than recipients of viral vector-based vaccines. Data such as these have been reflected in countries such as Brazil, where the level of acceptance for BNT162b2 (the only RNA vaccine currently approved in the country) is higher than those developed through other technological platforms, with lower reporting of post-vaccine AEs [286]. Health authorities are promoting critical awareness campaigns identifying the benefits of immunization with approved and safe vaccines. Large clinical (phase 3 trials) and real-life studies (after EUA) have shown the safe and effective profile of RNA-based vaccines in protecting against COVID-19, and the data-based incorporation of RNA-based vaccines in national immunization programs represents an important step to increase vaccine acceptance levels.

## 5. Conclusions

Despite being studied over the past few years and having initial studies conducted in the context of cancer vaccines [126,241,287], RNA-based vaccines first became available on a population level in response to the SARS-CoV-2 pandemic, highlighting the importance of having a highly safe, flexible, and scalable vaccine available for the prevention of not only for epidemic outbreaks, but also important diseases such as cancer, autoimmune diseases, and allergic disease. In addition, the many advantages of this technology when compared to conventional approaches, such as allowing the delivery of the genetic material directly to the cells, without the need to administer exogenous antigens, high immunogenicity, and production in a cell-free environment, allows RNA-based vaccines to have good potential in the future of biopharmaceuticals. Therefore, there is an increased interest from the industry in the development of this technological platform.

In the context of COVID-19, it is important to highlight not only the rapid development and availability of mRNA vaccines but also their high efficacy. Thus far, clinical trials and the subsequent expansive use of COVID-19 vaccines demonstrate not only the safety but also the efficacy of the RNA vaccine platforms. As demonstrated in clinical studies, mRNA vaccines, particularly Pfizer’s and Moderna’s mRNA vaccines, have been shown to be highly safe and effective. However, it is important to mention that, even with high efficacy, such vaccines need to be applied in two doses, and the application of a booster dose (third dose) is already being considered for risk groups (the elderly and people with immunodeficiencies). A promising alternative to resolve this issue is the application of saRNA vaccines, which, thanks to their self-replicative capacity, have the potential to confer an immune and long-lasting response with just a single dose. However, clinical immunogenicity data for these vaccines are not yet available.

It is important to highlight that many strategies need to be to evaluated when developing RNA-based vaccines. Among them are the delivery technologies applied to it since naked RNA cannot enter cells due to its intrinsic properties. Knowledge of the delivery platform composition is especially important since it impacts the formulation stability, the ability to induce an immune response, decreased reactogenicity, and the efficacy of RNA delivery. LNPs are the most used platform, and their composition provides a solid structure that protects RNA from degradation. It is also important to note that the components of a vaccine directly influence the planning and costs of manufacturing, key factors for making the immunizer available.

Despite the rapid response capability and flexibility of RNA vaccine technology demonstrated as these vaccines have become central to efforts to contain SARS-CoV-2, tied to their high safety and efficacy, RNA-based vaccines face significant challenges, mostly related to the scale of production, supply chains, and long-term efficacy, the latter of which is accentuated with the emergence of new variants. Moreover, RNA-based vaccines still face skepticism and strengthening anti-vaccine movements. However, thanks to its versatility, RNA technology is changing the vaccine industry and providing hope that it may be used to generate candidates with the potential to protect against a wide variety of cancers and infectious diseases [65].

## Figures and Tables

**Table 1:** Clinical trials with RNA-based vaccines against cancer. Data collected from the studies registered at ClinicalTrials.gov as of 8 August 2021.

Sponsor	NCT-Number	Country	Vaccine Type	Targets	Trial Phase	Status	Reference
Argos Therapeutics	NCT00087984	USA and Canada	DCs electroporated with autologous tumor mRNA	Metastatic renal cell carcinoma	1/2	Completed	NA
	NCT00664482	USA		Pancreatic cancer	Not Applicable		
University Hospital Tuebingen	NCT00204516	Germany	Naked mRNA	Melanoma	1/2	Completed	NA
Radboud University	NCT00228189	Netherlands	DCs electroporated with tumor-associated antigen mRNA	Colorectal cancer	1/2	Completed	[84]
Medigene AG	NCT02405338	Norway	Autologous DCs with tumor-associated antigen mRNA	Acute myeloid leukemia	1/2	Completed	[85]
BioNTech RNA Pharmaceuticals	NCT02035956	Austria and Germany	Naked RNA	Melanoma	1	Completed	[86]
Universitair Ziekenhuis Brussel	NCT01302496	Belgium	DCs electroporated with tumor-associated antigen and TriMix (CD70, CD40L, TLR4) mRNA	Melanoma	2	Completed	[87]
Herlev Hospital	NCT00978913	Denmark	DCs loaded with tumor-associated antigen mRNA	Breast cancer, melanoma	1	Completed	[88]
	NCT01446731			Prostate cancer	2		[89]
Duke Cancer Institute	NCT01890213	USA	Alphavirus replicon encoding the tumor-associated antigen	Colon cancer	1	Completed	[90]
	NCT00003433		Antigen-RNA-pulsed DCs	IV colon cancer, liver metastases	1/2		NA
	NCT00003432			Breast cancer		Terminated (low accrual)	
University Hospital Erlangen	NCT01983748	Germany	DCs loaded with autologous tumor mRNA	Melanoma	3	Recruiting	NA
University Hospital, Antwerp	NCT02649582	Belgium	DCs electroporated with tumor-associated antigen mRNA	Glioblastoma	1/2	Recruiting	NA
Changhai Hospital	NCT03468244	China	Naked mRNA	Advanced esophageal squamous carcinoma, gastric, colorectal, and pancreatic adenocarcinomas	Not Applicable	Recruiting	NA
University of Campinas	NCT03083054	Brazil	DCs electroporated with tumor-associated antigen mRNA	Acute myeloid leukemia	1/2	Active, not recruiting	NA
Guangdong 999 Brain Hospital	NCT02808416	China	DCs pulsed with tumor-associated antigen mRNA	Brain metastases	2	Active, not recruiting	NA
	NCT02709616			Glioblastoma	1		
Life Research Technologies	NCT01456065	Hungary and Austria	DCs loaded with tumor-associated antigen mRNA	Ovarian cancer	1	Unknown	NA

DCs, dendritic cells; NA, not available; USA, United States.

**Table 2:** Clinical trials with RNA-based vaccines against infectious diseases. Data collected from the studies registered at ClinicalTrials.gov as of 8 August 2021.

Sponsor	NCT-Number	Country	Vaccine Type	Targets	Trial Phase	Status	Reference
Argos Therapeutics	NCT00672191	United States and Canada	DCs loaded with autologous viral antigen and CD40L mRNAs	HIV	2	Completed	[128]
Massachusetts General Hospital	NCT00833781	United States	Autologous DCs loaded with viral antigen mRNA	HIV	2	Completed	[129]
CureVac AG	NCT02241135	Germany	RNActive viral Ag mRNA	Rabies	1	Completed	[130]
Moderna Therapeutics	NCT03392389	United States	Lipid-nanoparticle-encapsulated, chemically modified viral antigen mRNA	Human Metapneumovirus and Human Parainfluenza	1	Completed	[131]
	NCT03014089			Zika			NA
	NCT03325075			Chikungunya			[132]
	NCT03382405			Cytomegalovirus			NA
	NCT03076385	Germany		Influenza			[133]
Fundacion Clinic per a la Recerca Biomédica	NCT02413645	Spain	Naked Viral antigen and TriMix (CD40, CD70 and IL2) mRNA	HIV	1	Completed	[134]
AlphaVax	NCT00440362	United States	Alphavirus replicon vaccine expressing a viral hemagglutinin protein	Influenza	1/2	Completed	NA
	NCT00439803		Alphavirus replicon vaccine expressing viral genes	Cytomegalovirus	1		[135]
	NCT00097838	United States, Botswana, and South Africa	Alphavirus replicon vaccine expressing viral protein	HIV			[136]

DCs, dendritic cells; HIV, human immunodeficiency virus; NA, not available.

**Table 5:** Effectiveness of approved RNA-based vaccines against COVID-19 from other studies (real-world conditions).

Evaluated Vaccine	Doses	Country	Participants	COVID-19 Outcomes	Effectiveness (%)	Reference
BNT162b2	1	England	156,930	Symptomatic infection	61	[187]
				Hospitalization	80	
				Deaths	85	
BNT162b2	1	Scotland	1,331,993	Hospitalization	91	[188]
BNT162b2 and mRNA-1273 (Spikevax)	2	USA	1212	Hospitalization	87.1	[189]
BNT162b2	2	Israel	6286	Symptomatic infection	61 (first dose) to 89 (second dose)	[190]
BNT162b2	2	Israel	596,618	Documented infection	46 (first dose) and 92 (second dose)	[191]
				Symptomatic infection	57 (first dose) and 94 (second dose)	
				Hospitalization	74 (first dose) and 87 (second dose)	
				Severe disease	62 (first dose) and 92 (second dose)	

**Table 6:** Components of the major RNA-based vaccine against COVID-19: characteristics of the mRNA or saRNA and antigen used and its delivery system composition.

Vaccine Name	mRNA or saRNA Construct	Antigen	Delivery Platform Composition	Diluent
mRNA-1273 (Spikevax)	N1-methylpseudouridine Modified 5′ CAP1 structure (m^7^GpppNmN) Codon optimization (GC-enriched sequence)	Full-length S protein with two proline substitutions (K986P and V987P) Wuhan-Hu-1 (GenBank: MN908947.3)	SM-102 Cholesterol DSPC PEG2000-DMG	Sodium chloride
BNT162b2	N1-methylpseudouridine Modified 5′ CAP1 structure Codon optimization	Full-length S protein with two proline substitutions (K986P and V987P) Wuhan-Hu-1 (GenBank: MN908947)	ALC-0315 (proprietary to Acuitas) ALC-0159 DSPC Cholesterol	Sodium chloride
CVnCoV	Modified 5′ CAP1 structure (m^7^GpppNmN) Codon optimization (GC-enriched sequence	Full-length S protein with two proline substitutions (K986P and V987P) Wuhan-Hu-1 (GenBank: YP_009724390.1)	Cholesterol DSPC PEG-ylated lipid Ionizable lipid (undisclosed/proprietary to Acuitas)	Sodium chloride
ARCT-021 (LUNAR-COV19)	N1-methylpseudouridine Modified 5′ CAP1 structure (m^7^GpppNmN) Codon optimization Self-replicating replicon of VEEV	Full-length, unmodified S protein Wuhan-Hu-1 (GenBank: YP_009724390.1)	Ionizable lipid (undisclosed) DSPC Cholesterol PEG2000-DMG	Sodium chloride
COVAC1 (LNP-nCoVsaRNA)	Modified 5′ CAP1 structure (m^7^GpppNmN) Self-replicating replicon of VEEV F318V amino acid substitution	Full-length S protein with two proline substitutions (K986P and V987P) and GGGGSGGGGS linker Wuhan-Hu-1 (GenBank: QHD43416.1)	Ionizable cationic lipid (undisclosed) Phosphatidylcholine (undisclosed) Cholesterol PEG-lipid (undisclosed)	Phosphate-buffered saline
ARCoV	Modified 5′ CAP1 structure (m^7^GpppNmN) Codon optimization	RBD region of S protein Wuhan-Hu-1 (GenBank: MN908947.3)	Ionizable lipid (undisclosed/proprietary to Acuitas) DSPC Cholesterol PEG-lipid (undisclosed)	Not reported
HDT-301 (LION/repRNA-CoV2S)	Modified 5′ CAP1 structure Self-replicating replicon of VEEV	Full-length, unmodified S protein Wuhan-Hu-1 (GenBank: MN908947.3)	DOTAP Squalene Span 60 Polysorbate 80 oleic-acid-coated iron oxide nanoparticles	Sodium citrate buffer

ALC-0159, 4-Hydroxybutyl)azanediyl)bis(hexane-6,1-diyl) bis(2-hexyldecanoate); ALC-0315, 4-Hydroxybutyl)azanediyl)bis(hexane-6,1-diyl) bis(2-hexyldecanoate); DOTAP, lipid 1,2-dioleoyl-3-trimethylam-monium-propane; DSPC, 1,2-distearoyl-snglycero-3 phosphocholine; PEG2000-DMG, 1 monomethoxypolyethyleneglycol-2,3- dimyristylglycerol with polyethylene glycol of average molecular weight 2000; Span 60, sorbitan monostearate; VEEV, venezuelan equine encephalitis virus.

## Data Availability

Data are contained within the article.
